# The interpersonal and intrapsychic functions of the pride expression

**DOI:** 10.1098/rstb.2025.0149

**Published:** 2026-09-24

**Authors:** Jessica L. Tracy, Zachary Witkower

**Affiliations:** ^1^ Department of Psychology, University of British Columbia, Vancouver, British Columbia, Canada; ^2^ University of Amsterdam, Amsterdam, The Netherlands

**Keywords:** emotion expression, pride, prestige, status, dominance

## Abstract

A large body of research suggests that pride is associated with a distinct, cross-culturally recognized and spontaneously displayed nonverbal expression. Given its apparent universality, this expression is likely to have evolved as a functional signal, benefitting both receivers and senders. Here, we focus primarily on the intrapsychic benefits received by those who express pride. We review research suggesting that the expression communicates an individual's deservedness of high status to others, and that it does so implicitly and across diverse cultural contexts. We then discuss the expression's function in the context of dual-strategies theory, which suggests that there are two distinct forms of high rank: prestige, based on earned respect, and dominance, based on threat of force. Studies suggest that the pride expression reliably and cross-culturally communicates prestige, but not dominance. Finally, we discuss functions of the pride expression that go beyond the display's signal value. In sum, the pride nonverbal expression appears to be a highly functional display that likely evolved to facilitate humans' adaptive responding to opportunities for social rank attainment.

This article is part of the theme issue ‘Mechanisms, development, phylogeny and functions of emotional expressions’.

## 1. Introduction

‘Of all the… complex emotions,’ Darwin [1] wrote, ‘pride, perhaps, is the most plainly expressed.’ He continued, ‘a proud man exhibits his sense of superiority over others by holding his head and body erect… He… makes himself appear as large as possible, so that metaphorically he is said to be swollen or puffed up…’ (p. 276).

When the empirical study of emotion expressions began, about a century after Darwin's observation, his comment on pride went largely ignored. For some time, researchers focused exclusively on emotions that could be expressed by the face alone. Eventually, scholars returned to Darwin's interest in fully embodied expressions, and studies were conducted which supported his observation. When individuals feel pride, they show it through a distinct set of nonverbal behaviours, which include the body and the face. These behaviours, in turn, serve an important function. They relay information about the proud individual's social rank, making pride a signal that serves both interpersonal and intrapsychic functions. The high-rank message sent by pride displays fits well with the broader emotional experience of pride, which involves subjective feelings centred on accomplishment, confidence and self-worth [2]. Here, we review the accumulated research evidence for a universally recognized and displayed pride expression along with its adaptive functions.

## 2. The prototypical pride expression

**Figure 1.**
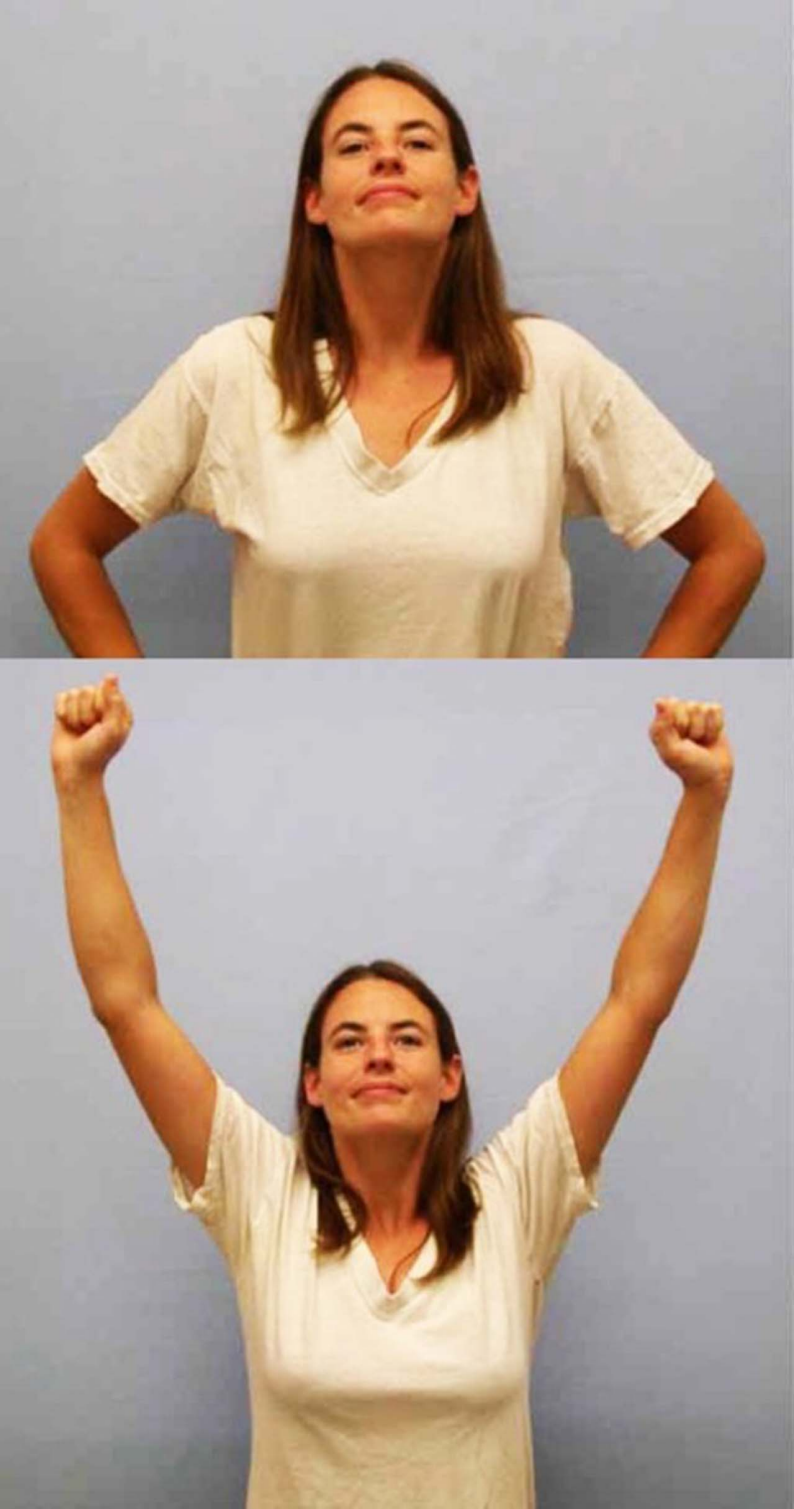
The prototypical pride expression.

Historically, the presence of a distinct, cross-culturally recognized non-verbal expression was considered the gold standard for determining whether an emotion is likely to be a biologically based and evolved feature of human nature (e.g. [3,4]). Although pride was not included in the pantheon of emotions originally thought to meet this criterion (e.g. [5]), studies conducted over the past two decades demonstrate that pride is, in fact, associated with a reliably recognized nonverbal expression [6,7]. The prototypical pride expression consists of an expanded and upright posture, head tilted slightly upward (about 20 degrees), a small smile, and arms extended out from the body, either akimbo with hands on the hips or raised above the head with hands in fists (see figure 1). The expression is highly recognizable when displayed by both men and women [6,7] and, although the original studies supporting these findings were conducted on American undergraduates, pride recognition was subsequently replicated among a community sample in Bologna, Italy [8] and several small-scale traditional societies [8,9].

Recognition of the pride expression in educated North American samples ranges from 80 to 90%—comparable to rates found for other well-studied emotion expressions (e.g. anger, sadness) [6,7,10–12]. Like those other expressions, pride can be recognized quickly and efficiently (i.e. even under cognitive load) from a single image [13], suggesting that recognition occurs through an automatic cognitive process. Developmental research has documented early onset of recognition; children first demonstrate accurate labelling of pride displays at 4 years of age, around the same time at which they can accurately label most other emotion expressions [14]. However, in contrast to basic emotion expressions like happiness, anger and fear, pride expressions require visible bodily behaviour (expansive posture) or, at least, head movement tilting upward, to be accurately recognized [6,15]. This distinction might be indicative of the expression's phylogenetic history; if the display evolved from non-human primate dominance displays, which are conveyed largely through the body, it would make sense that the expression retains that core bodily expansiveness component even in humans [4].

Importantly, the pride expression is not only reliably recognized but also spontaneously displayed in pride-eliciting situations: when individuals experience success. Behaviours such as head tilted upward, erect posture and arms stretched upward and out from the body have been documented among preschool children who won a fight [16], high-school students who performed well on a class exam [17] and children as young as 3 years old in response to task success [18–20]. By the age of three, it seems, individuals reliably experience pride in their achievements and display components of the expression accordingly.

### (a) Cross-cultural generalizability

Evolutionary accounts of emotion expressions typically demand evidence of reliable recognition across diverse cultural contexts, including populations with limited exposure to Western cultural influences, to ensure that displays are not merely culture-specific gestures. The logic underlying this approach—often referred to as the maximally divergent population test of universality [21,22]—is that individuals from culturally and geographically isolated societies are unlikely to have learned Western emotion expressions through cross-cultural transmission. Given this fact, the documented ability of these individuals to recognize a given display provides evidence that recognition of that display does not depend solely on cultural learning. Instead, such findings would be consistent with the suggestion that the display is an evolved feature of human psychology, and is therefore likely to be universal.

Several studies have examined recognition of pride across such diverse cultural contexts, and although rates tend to be somewhat lower in non-Western than Western cultures (as is the case for all emotion expressions originally documented in Western nations; see [23]), reliable recognition of pride has been documented in two highly isolated, traditional small-scale societies in disparate parts of the world: Burkina Faso and Fiji [8,9]. Both studies were conducted among individuals who had rarely, if ever, left their local communities and had no exposure to Western media such as magazines, films or television. In the Burkina Faso study, participants could not read or write, communicate in any language other than their local dialect, nor recognize the faces of a small set of internationally famous celebrities (e.g. the US president at the time, Tom Cruise, Michael Jordan). The Fijian study sampled members of a society with highly prescribed status rules (i.e. status is completely determined on the basis of gender and family), resulting in strong cultural norms against any kind of display that might convey a person's belief about their own power, status or pride. The finding that both samples of individuals nonetheless showed reliable recognition of pride expressions provides some of the strongest evidence to date for the expression's universality.

Furthermore, the tendency to spontaneously display pride in response to success also generalizes across cultures. Tracy & Matsumoto [24] coded the nonverbal behaviours shown by judo athletes in the 2004 Olympic Games from close-up, high-definition photographs taken immediately after matches were completed. Athletes from 36 nations were substantially more likely to display each of the behaviours associated with the prototypical pride expression when they won their match compared to when they lost. This pattern was replicated across male and female athletes, and across individualistic countries such as the United States, Canada and Estonia, more collectivistic countries such as China and Iran, countries with ‘secular-rational’ values such as Belgium and Finland and countries with more ‘traditional-religious’ values such as Ireland and Poland.

Most importantly, a follow-up study used the same method to examine nonverbal behaviours displayed by an international sample of blind judo athletes participating in the Paralympic Games [24]. Results were replicated, even among a smaller sub-sample of congenitally blind athletes—individuals who could not have learned to show pride through visual modelling. Again, winners were more likely to spontaneously display several components of the prototypical pride expression compared to losers.

Taken together, these findings strongly suggest that the pride expression is likely to be an innate behavioural response to success, and establish pride as an adaptation by supporting multiple falsifiable hypotheses (see [25]). As is the case for many phenomena in the study of human evolution, less parsimonious alternative explanations remain possible. However, it is difficult to explain why the pride expression is recognized consistently and robustly, including by individuals who could not have learned it through cross-cultural transmission, and why it is reliably and spontaneously displayed in pride-eliciting situations, including by individuals who have never seen others show it, if it is not a biologically ingrained human universal.

### (b) Cultural and contextual differences in pride

Nonetheless, cultural and situational factors influence when the pride expression is displayed and when it is suppressed. Displaying pride draws attention to one's achievements, which can promote social status but can also lead to negative social outcomes. For example, observers tend to envy those who show pride, and also come to like them less and view them as arrogant [26,27]. As a result, pride expressions are often suppressed in contexts where individuals expect the expression or their success to elicit negative evaluations [28,29]. People also tend to suppress pride expressions when their achievement occurs in a domain that is especially relevant to observers [28], given that displays of pride in these contexts would be particularly envy evoking [30]. Furthermore, some theoretical accounts suggest that the pride expression is regulated by a magnitude-matching system that calibrates displays to the value audiences are expected to place on the achievement [31]. This account might explain why pride is strategically suppressed when observers are likely to devalue the success or experience envy.

Supporting this theory, van Osch *et al.* [32] tested whether national and Olympic athletes from the US and China differ in the extent to which they display pride upon receiving gold medals. When Olympic gold medallists outperformed outgroup members (i.e. athletes from other countries), there was little-to-no difference between Chinese and American athletes in the pride expressions they displayed, replicating Tracy & Matsumoto's [24] findings. However, when athletes outperformed fellow ingroup members in national competitions (i.e. athletes from their own country), Chinese athletes expressed less pride than did Americans. Importantly, the finding that Chinese athletes downregulated their pride expressions when outperforming an ingroup member, but not one from an outgroup, suggests that this pattern is unlikely to reflect a cultural difference in the propensity to experience pride in response to success. Instead, it points to culturally contingent norms governing the display of pride, particularly in contexts where expressing pride might threaten ingroup harmony.

These cultural differences are also relevant to another critical feature of any evolved signal: that it be costly. Theoretical accounts suggest that to evolve as a reliable (i.e. trustworthy) signal, a display must either not be fakeable, or be costly when faked [33,34]. For non-human animals, costs typically come in the form of wasted metabolic resources (as in the plumage that constitutes a male peacock's tail), but for humans these costs can be socially imposed. Pride displays are easily faked, so those who show them when they are not warranted are likely to face social costs, in the form of group rejection.

Indeed, proverbs across many cultures hold that those who express pride are subsequently viewed in a negative light, at least in certain contexts [35]. In fact, both Fijian villagers and Canadian undergraduates tend to judge pride displayers less positively than those who show happiness [9,36]. Wubben *et al.* [37] also found that pride displays shown when the emotion is not clearly warranted are judged as hubristic and, as a result, lead to inferences that the expresser is behaving in an anti-social manner. In Spain, a culture that tends to be somewhat collectivistic, pride displays are seen as arrogant, and individuals report greater efforts to control their pride expressions compared to participants from the Netherlands, a more individualistic nation [38].

Considering these costs, openly displaying pride may be a risky strategy; showing it without clear evidence of deservedness can lead to gains in status but losses in social inclusion or respect. The threat of social disapproval or outright rejection raises the cost of pride displays and deters would-be fakers. However, the social norms that prohibit inappropriate pride displays likely vary across cultures, in ways that may track cultural differences in status seeking and conferral.

## 3. Functions of the pride expression

The findings reviewed above suggest that the pride expression is likely to be a universal and potentially evolved feature of the human emotional and behavioural repertoire. From this perspective, the expression can be understood as an adaptation that affords benefits to individuals who succeed in valued domains—the situations that tend to evoke pride. Indeed, several scholars have argued that the pride expression evolved to help individuals transform culturally valued achievements into higher social status (e.g. [39,40]). Across species, adaptive benefits accrue to those who effectively send and receive signals of high status through readily identifiable nonverbal displays. These individuals tend to receive greater social influence and attention [41,42], a greater allocation of potentially scarce resources [43], higher quality mates (e.g. [44]) and deference [45]. Although many successes occur in the presence of observers, throughout humans' evolutionary history many did not (e.g. a hunter returning home after a large kill), making it adaptive to nonverbally communicate one's success to others who did not witness it.

More broadly, when considering the functions served by emotion expressions, it is useful to consider a distinction originally made by Darwin [1], who proposed that emotion expressions evolved to serve two classes of functions: (i) a physiological function that prepares an organism to respond adaptively to environmentally recurrent stimuli and (ii) a signalling function that communicates critical social information. Subsequent researchers (e.g. [5,39,46,47]) further developed this account, arguing that internal physiological regulation was likely the original adaptive function of emotion expressions, which later evolved to serve communicative functions.

Although we might be tempted to map these two kinds of functionality onto an intrapsychic/interpersonal distinction, that is probably an oversimplification. The original physiological functions that Darwin discussed are unlikely to be the only benefit expressers receive from emotion expressions, given that they are regularly displayed in exaggerated, highly prototypic and visually obvious ways, and in response to evolutionarily recurrent situations that, in some cases, seem unrelated to those of their original physiological function (e.g. disgust shown in response to morally reprehensible acts [39,46,48]).

Another way to understand these dual functions is to draw on a distinction evolutionary biologists make between cues and signals. A cue provides information gleaned as a byproduct of something that serves an alternate adaptive purpose; for example, chewing is a reliable cue that someone is eating, but it did not evolve to communicate that information [33]. Signals, on the other hand, evolved specifically for the purpose of communication; for example, peacock plumage evolved as a hard-to-fake signal of mate quality [49]. In their two-stage model, Shariff & Tracy [39] hypothesized that emotion expressions began as cues—providing information about expressers' internal states but not existing for that reason—but eventually transformed, in both form and function, to become signals. In other words, over the course of evolutionary history, the function of expressions itself evolved. As recognizing the internal states of other animals yielded fitness-positive consequences, the facial and bodily behavioural components of certain emotions came to cue those emotional states to observers. As social interaction became more possible and even vital for many species, the adaptive value of these expressions may have shifted toward communication. As a result, the nonverbal behaviours associated with distinct emotions likely underwent ritualization: a process of change well researched in evolutionary zoology whereby an animal's nonverbal displays become exaggerated, more visible, distinctive and/or prototypic in order to function as reliable and effective signals [47].

For emotion expressions, this shift from cue to signal can be thought of as their second stage of evolution—a paradigmatic example of exaptation, the evolutionary process whereby a feature that evolved for one reason gradually morphs to serve a secondary adaptive function. As a result of ritualization, emotional expressions became the highly recognizable displays we regularly see today. Although for certain expressions some degree of original physiological functionality may be retained (e.g. widened eyes that occur with the fear facial expression function to expand one's visual scope [50]), the primary purpose of emotional expressions in contemporary human life, and humans' primary preoccupation with them, has more to do with their capacity to quickly and nonverbally communicate socially significant information. That communication, in turn, provides both interpersonal benefits to observers and intrapsychic benefits to expressers.

In this view, an expression that benefits its expresser can be said to be serving an intrapsychic function even if those benefits are accrued largely or only by virtue of others recognizing or responding to the expression. By contrast, an expression that benefits onlookers more clearly serves an interpersonal function. In the case of pride, the expression's widespread recognizability provides interpersonal benefits to observers, who acquire important information about others in their social sphere who are likely to have demonstrated a socially valued success and thus should be treated as a high-status social model. It also provides intrapersonal benefits to expressers, who are conferred status by observers when they display this recognizable signal. Although this latter benefit occurs as a result of interpersonal communication, we nonetheless consider it to be at least somewhat intrapersonal, given that the recipient of the benefit is the same person who displayed the expression.

In fact, a large body of research suggests that individuals who display pride receive adaptive benefits from their display, in the form of increased social rank. Studies using the Implicit Association Test (IAT [51]) to measure the speed with which participants respond to pride expressions when paired with high versus low status concepts show that observers automatically perceive pride displays as conveying high status [36]. Subsequent work showed that the status signal sent by pride displays is powerful enough to override contradictory low-status cues in the environment, such as clothing or social roles [52]. Moreover, studies examining status associations with versions of the expression that include only components that expand the target's appearance (e.g. arms outstretched) determined that the automatic association between the comprehensive pride display and high status could not be attributed to the fact that the expression makes the individual appear larger.

The most convincing evidence for this account of pride displays as evolved status signals is the finding that the automatic tendency to perceive the expression as conveying high status generalizes across diverse populations. Tracy *et al.* [9] replicated the finding of implicit status associations in a highly isolated, traditional small-scale society on a remote island in Fiji. As noted above, Fijian cultural rules prohibit individuals from engaging in nonverbal behaviours that might communicate their belief that they deserve increased status, making Fiji a tough test of the universality of the pride expression status signal. Despite these cultural norms, and the fact that the population sampled had little-to-no contact with the rest of the world, results were largely convergent with those of North American university students: in both groups, pride displays were strongly implicitly associated with high-status concepts.

## 4. Dual-strategies theory: dominance and prestige

Although a substantial body of research suggests that pride expressions function to signal high status, status is itself multifaceted. Numerous lines of work from anthropology, evolutionary biology and psychology converge to suggest that humans use two distinct strategies to attain high social rank or status. Specifically, individuals can effectively attain higher rank through prestige, which involves the demonstration of valued knowledge and expertise to elicit earned respect, admiration and freely conferred deference; and dominance, which involves the use of aggression and intimidation to instil fear and forced compliance ([41,53–56]; but see [57,58]).

Prestige and dominance are distinct and largely independent, yet both are effective strategies for attaining higher rank, enabling individuals to influence others and ascend social hierarchies (e.g. [41,59,60]). Both the distinctiveness and effectiveness of these strategies have been well-documented across human populations, including WEIRD (Westernized, Educated, Industrialized, Rich, Democratic; [61]) societies in North America [41] and non-WEIRD, small-scale, traditional communities around the world [54,60,62,63]. Notably, every study that has made efforts to measure both forms of high rank and to circumvent issues of collinearity between often-strongly related high-status concepts has found that dominance and prestige reliably emerge as distinct predictors of social rank or influence [57]. This pattern holds across male and female groups, suggesting that both men and women use tactics of dominance and prestige, and both strategies are effective across gender [41]. Thus, although pride expressions function as widely recognized, cross-cultural and automatic signals of status [9,36], the dual-strategies framework raises an important question: does pride signal prestige, dominance or both?

### (a) Pride expressions signal prestige

Converging evidence across studies suggests that the pride expression may have evolved to communicate an individual's prestige—and not dominance—to others. First, prestigious leaders predominantly acquire their power and influence by allowing others to copy and learn from them. Studies suggest that pride expressions may play a critical role in this process.

Martens & Tracy [64] examined whether financially motivated observers would choose to copy answers to difficult trivia questions provided by another group member (actually a confederate) if that individual displayed pride. Across studies, participants copied the answers of pride-displaying confederates substantially more frequently than those of confederates displaying neutral, shame or happy expressions.

Other studies have more systematically examined the specific nonverbal behaviours that promote perceptions of prestige. Witkower *et al.* [65] separately manipulated the three critical components of the prototypical pride expression: head tilt (upward versus downward versus neutral), posture (expansive versus neutral) and smiling (slight smile present versus absent) and assessed effects on prestige judgements (see also [66,67]). The configuration of behaviours constituting the pride expression—head tilted upward, expansive posture and slight smile present—consistently elicited the strongest prestige perceptions, regardless of whether the behaviours were manipulated independently of one another or in combination. Importantly, this configuration did not lead to perceptions of dominance, indicating a distinctive link between pride expressions and prestige. This finding was replicated across displayer and perceiver gender, and multiple pre-registered samples.^1^

Subsequent research found that the pride expression is reliably identified as conveying prestige among the Mayangna, a small-scale traditional society in the remote Bosawás Biosphere Reserve in Nicaragua [63]. Participants from this community had limited formal education and minimal exposure to Western media or any cultural influences outside of their own community. Nonetheless, they reliably identified proud targets as prestigious (i.e. ‘likely to be a leader because he/she is accomplished and admired, and shares useful knowledge with others’) and not dominant (‘likely to be a leader because he/she is willing to use aggression and intimidation to get his/her way’). Given the cultural isolation of this population, this finding provides strong support for the suggestion that the pride expression's connotation of prestige is not a product of cultural learning, but instead a cross-cultural feature of human communication. This signalling function of the expression is likely to entail both interpersonal and intrapsychic benefits, as observers across cultures learn from a quick signal whom they should follow, copy and defer to; and expressers acquire an increase in social rank and influence by virtue of others' perception of their prestige.

Supporting this account, other studies have shown that spontaneous displays of pride, in real-world and in-lab contexts, lead to perceptions of prestige and, as a consequence, increases in displayers' social rank. Witkower *et al.* [65] observed participants work in teams to complete a collaborative group task. Based on nonverbal behavioural coding, participants who displayed the full suite of behaviours characteristic of the prototypical pride expression—expansive posture, upward head tilt and smiling—were perceived by their teammates as more prestigious. Moreover, these individuals had greater influence over their peers’ decision-making—that is, greater power and social influence—as evidenced by both participant reports and independent observer ratings. Although we cannot be sure that these individuals were subjectively experiencing pride while displaying behaviours consistent with the expression, analyses confirmed that observers relied on these pride-typical expressions to guide their judgements of targets and grant them social rank.

Research in economic decision-making also supports the conclusion that pride displays communicate prestige. In negotiations, those who show pride elicit greater trust and more generous offers from interaction partners [69]. By contrast, in more altruistic contexts, such as when requesting charitable donations to support one's small business venture, pride displays can backfire. Needy individuals who displayed pride when soliciting donations were found to receive significantly less financial support, rather than more [70]. Although displays indicating prestige might be expected to communicate an individual's competence, and therefore high likelihood of repaying their benefactor, these displays may also communicate low need, which can undermine the necessity for charity[70].

Although no neuroimaging studies have directly assessed overlap in the neural basis of perceptions of pride and social rank, there is evidence for potentially significant functional overlap across several key regions. Cortically, the medial prefrontal cortex (mPFC) and the posterior superior temporal sulcus (pSTS) are sensitive to both pride-related experiences and the perception of social hierarchy ([71–73]; see also [74–76]). Subcortical involvement is also evident; the amygdala responds to cues of pride and dominance-based rank [72,77], whereas the ventral striatum is consistently recruited by pride- and prestige-based rank [72,76,78,79]. This is an area where additional research is much needed.

### (b) Pride expressions and intrapersonal feelings of prestige

As noted above, the Two Stage Model of emotion expressions' evolution suggests that facial expressions of emotion originally evolved to serve intrapsychic physiological functions [39]. However, the more social, self-conscious emotion expressions, which tend to involve the body as well as the face (e.g. [80]), may have taken a different phylogenetic course. These emotions may have initially evolved to serve a direct communicative function, meaning that, in contrast to expressions like anger or happiness, the human pride expression may not be a ritualized version of a more ancient cue. Instead, scholars have argued that the pride expression's phylogenetic origins lie in ancient non-human dominance displays, which often involve bodily and head movements similar to human displays of pride, and which are thought to have served a communicative function. High-ranking chimpanzees, for example, have been observed to show ‘inflated’ or ‘bluff’ displays after defeating a rival and prior to an agonistic encounter; these include behaviours such as arms raised and body expanded [35,81]. The chest-beating intimidation displays of gorillas [82], and the ‘strutting confident air’ characteristic of dominant catarrhine monkeys [83] also share behavioural similarities with components of the human pride expression, such as the critically involved expanded chest or posture [7]. In addition to these mammals, expansive nonverbal behaviours are used to signal high rank in birds [84], arachnids [85], reptiles [86,87] and fish [88].

Nonetheless, there is evidence to suggest that the pride expression additionally serves a physiological or psychological intrapsychic function for humans today. Tracy & Shariff [39] hypothesized that the expression might have originally evolved to boost testosterone and—given the expansive chest and postural components—increase the expresser's lung capacity, which could be valuable in preparation for an agonistic encounter. Although no evidence exists to support the latter hypothesis, Carney *et al.* [89] found that individuals who posed expressions of pride (which these authors called ‘power poses’) showed increases in testosterone, a hormone known to promote aggression. This effect, however, has been subjected to numerous replication attempts, which, overall, failed to reliably reproduce the result [90]. Yet these studies also found that displaying and maintaining open and expansive nonverbal behaviours (i.e. ‘power posing’) reliably increases self-reported feelings of power [89], as well as confidence and a tendency to take action [91,92]. Multiple preregistered replications using various meta-analytic techniques have converged on a robust, small-to-moderate sized overall effect [93]. An even more comprehensive meta-analysis of this literature, which included both pre-registered and non-pre-registered studies, provides further support for the association between posing the pride expression and heightened feelings of power [94]. Displaying pride therefore seems to increase feelings of power, but the biological mechanism underpinning this effect remains elusive.

Although no research has directly examined whether displaying pride-like behaviours increases self-reports of prestige specifically, power posing has been shown to increase positive self-attitudes, self-esteem and confidence [95,96]—all outcomes that are conceptually more aligned with prestige than dominance. Displaying components of the pride expression thus seems to robustly elicit intrapersonal feelings of status, in a form that may be more consistent with prestige than dominance.

### (c) Pride expressions and dominance

The association between pride expressions and dominance is, by contrast, much more tenuous. Interpersonally, pride displays do not elicit conferrals of dominance from observers [65]. However, expansiveness and an upward head tilt—components of the pride display—do reliably convey dominance among North American samples, albeit not as strongly as prestige, when both are tested [65,97–99]. Smiles, by contrast, substantially undermine dominance perceptions [65,100]. Moreover, although an upward head tilt communicates dominance to a certain extent, a downward head tilt, which decreases perceptions of pride [6,7], is a far stronger signal of dominance [65–67,101]. Furthermore, the specific forms of expansiveness that convey prestige may differ from those that convey dominance. In exploratory analyses, Witkower *et al.* [65] found that prestigious individuals engaging in actual rank contests displayed more restrained postural expansion consistent with the pride expression—slightly pressing out their chests and extending their torsos—whereas dominant individuals in these situations exhibited more overt, space-occupying behaviours, such as spreading their arms outward and taking up more physical space (see also [96,102]).

Taken together, pride and its prototypical expression reliably function as a mechanism for signalling and thereby attaining prestige, but not dominance. Instead, dominance is conveyed through a set of behaviours that are distinct—and in some cases contradictory—to the prototypical pride expression. Across controlled experiments and a real-world political contest, nonverbal displays featuring a downward head tilt, a neutral facial expression and open, expansive postures were found to reliably elicit perceptions of dominance [65]. Furthermore, this configuration of nonverbal behaviours is reliably recognized as dominance and not prestige among the Mayangna of Nicaragua, as well as North American children as young as 2–3 years old [63]. These findings indicate the widespread generalizability of the dominance nonverbal display and, much like the pride expression, establish it as a core feature of human communication.

## Data Availability

This article has no additional data.

## Footnote

^1^
Notably, changing the head tilt to shift downward instead of upward changes perceptions of the expression dramatically, such that even a smile paired with a downward head tilt is judged as anti-social and dominant, rather than proud or prestigious [66,68].

## Theme

One contribution of 19 to a theme issue ‘Mechanisms, development, phylogeny and functions of emotional expressions’.
